# Tibialis posterior in health and disease: a review of structure and function with specific reference to electromyographic studies

**DOI:** 10.1186/1757-1146-2-24

**Published:** 2009-08-19

**Authors:** Ruth Semple, George S Murley, James Woodburn, Deborah E Turner

**Affiliations:** 1 Division of Podiatric Medicine and Surgery, School of Health, Glasgow Caledonian University, Glasgow, UK; 2 Department of Podiatry, Faculty of Health Sciences, La Trobe University, Bundoora, Australia; 3 Musculoskeletal Research Centre, Faculty of Health Sciences, La Trobe University, Bundoora, Australia; 4 HealthQWest Research Consortium, School of Health, Glasgow Caledonian University, Glasgow, UK

## Abstract

Tibialis posterior has a vital role during gait as the primary dynamic stabiliser of the medial longitudinal arch; however, the muscle and tendon are prone to dysfunction with several conditions. We present an overview of tibialis posterior muscle and tendon anatomy with images from cadaveric work on fresh frozen limbs and a review of current evidence that define normal and abnormal tibialis posterior muscle activation during gait. A video is available that demonstrates ultrasound guided intra-muscular insertion techniques for tibialis posterior electromyography.

Current electromyography literature indicates tibialis posterior intensity and timing during walking is variable in healthy adults and has a disease-specific activation profile among different pathologies. Flat-arched foot posture and tibialis posterior tendon dysfunction are associated with greater tibialis posterior muscle activity during stance phase, compared to normal or healthy participants, respectively. Cerebral palsy is associated with four potentially abnormal profiles during the entire gait cycle; however it is unclear how these profiles are defined as these studies lack control groups that characterise electromyographic activity from developmentally normal children. Intervention studies show antipronation taping to significantly decrease tibialis posterior muscle activation during walking compared to barefoot, although this research is based on only four participants. However, other interventions such as foot orthoses and footwear do not appear to systematically effect muscle activation during walking or running, respectively. This review highlights deficits in current evidence and provides suggestions for the future research agenda.

## Introduction

The tibialis posterior (TP) muscle has a vital role during gait; via multiple insertion points into the tarsal bones it acts as the primary dynamic stabiliser of the rearfoot and medial longitudinal arch (MLA) [1,2]. The significance of TP function is evident when the muscle and tendon are dysfunctional, whereby stability of the foot is compromised and is associated with a progressive flatfoot deformity [3]. Prevalence data on TP tendon dysfunction (TPTD) is lacking, however it has been recognised as a painful and disabling condition affecting multiple patient groups [4-6] and is frequently encountered in podiatric practice. Assessing the function of the TP muscle and tendon can be determined through careful clinical examination including techniques such as manual muscle testing and the single heel rise test [7,8]. Clinical examination can be supplemented with more specialist modalities including muscle function magnetic resonance imaging (MRI) [9], ultrasound [10], electromyography (EMG) [11,12] and gait analysis [11,13,14]. The purpose of this paper is to provide an overview of TP muscle and tendon anatomy and to review current evidence that describes normal and abnormal tibialis posterior muscle activation during gait based on EMG.

## Anatomy and Function

**Figure 1:**
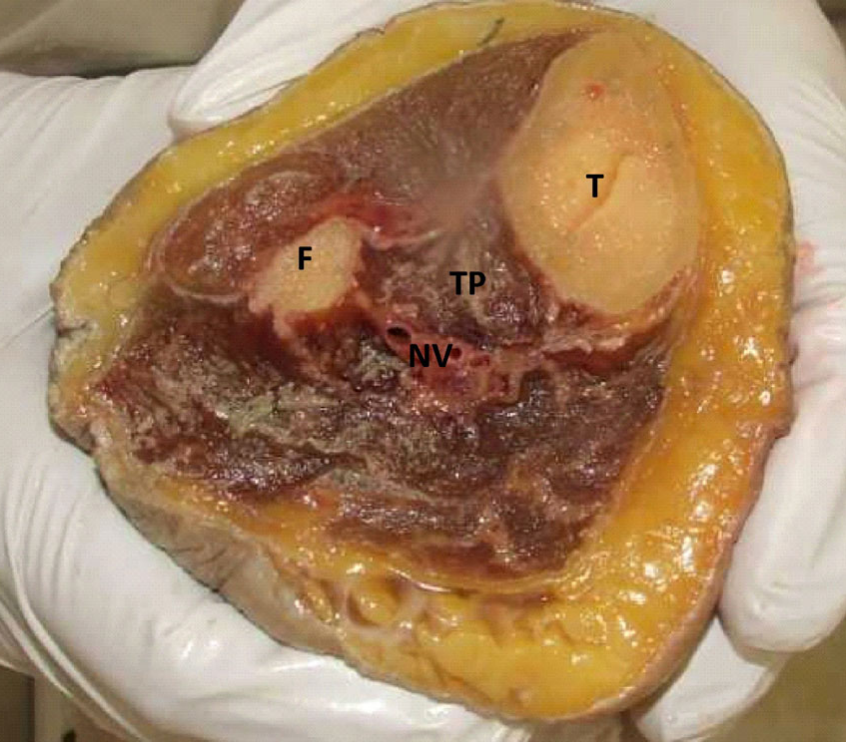
**Cross sectional anatomy**. Cross section of cadaver limb, taken 10 cm distal to the knee joint, indicating origin and depth of the TP muscle and inaccessibility for surface EMG investigation; tibia (T), fibula (F), tibialis posterior (TP) and neurovascular bundle (NV).

**Figure 2:**
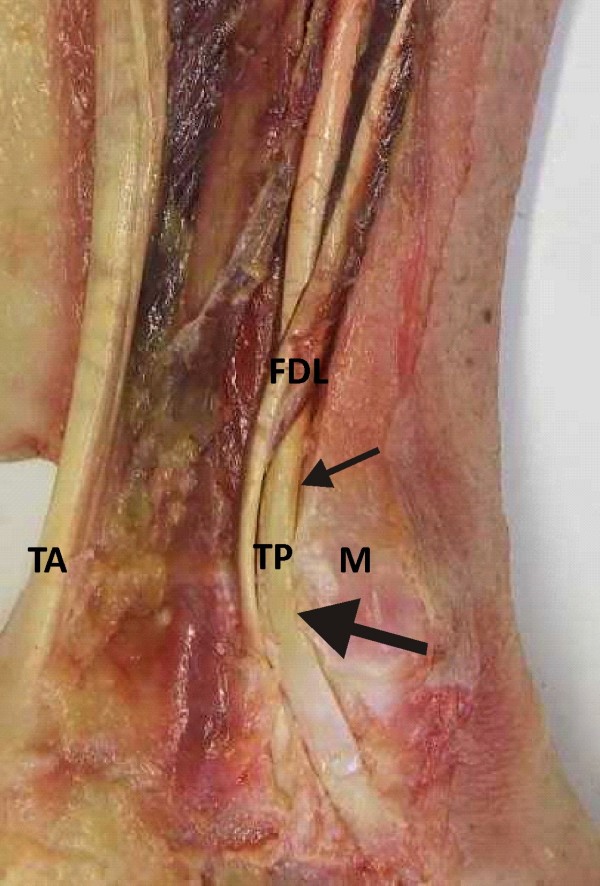
**Gross anatomy of retromalleolar region**. Gross anatomy of retromalleolar region indicating flexor digitorum longus tendon (FDL), tibialis posterior tendon (TP), medial malleolus (M) and tendo Achilles (TA). Small arrow indicates rounded TP tendon proximally and large arrow indicates the flattened area of tendon in retromalleolar region.

The TP muscle is contained within the deep posterior compartment of the lower limb, arising from the adjacent posterior surfaces of the tibia, fibula and interosseus membrane (Figure 1). The tendon forms in the distal third of the leg and changes direction to enter the foot where it passes acutely behind the medial malleolus. In this region the tendon flattens (Figure 2) and the tissue structure changes; exhibiting an increased presence of fibrocartilage [15,16] and an avascular region [17,18]. The tendon is enclosed within a synovial sheath and is held firmly in place by the flexor retinaculum which forms the roof of the tarsal tunnel. The location of the TP tendon relative to the axes of the subtalar and ankle joints facilitates inversion and plantarflexion respectively. Tibialis posterior is described as the most powerful supinator of the hindfoot as a result of the large inverter moment arm acting on the subtalar joint [19,20].

The TP tendon has multiple insertions within the foot, dividing into three main components: (i) anterior; (ii) middle; and (iii) posterior [21-23]. The anterior component is the largest and extends to the navicular tuberosity; it is reported to contain a fibrocartilaginous or bony sesamoid at this site. The sesamoid functions to provide a pressure absorbing or gliding mechanism and was found in 23% of 348 adult feet [24]. The middle and posterior components extend to the remaining tarsal bones, the middle three metatarsals and the flexor hallucis brevis muscle. The complex anatomy of the insertion sites function to stabilise the MLA. Variations of the insertion have been reported in the literature [21,22]; however the structural and functional significance of these variations are unknown.

### Tibialis posterior intramuscular EMG

The most common modality used to quantify TP muscle activation is via EMG recorded with intramuscular electrodes. The advantage of using EMG over other modalities (such as MRI and ultrasound) is the ability to investigate muscle activation simultaneously with dynamic weightbearing tasks such as walking. However, due to the deep location of the muscle within the posterior compartment of the leg, surface electrodes cannot record TP EMG activity without signal cross-talk from various superficial muscles (Figure 1). Therefore, one disadvantage of assessing TP with EMG is the requirement to use invasive intramuscular electrodes, which occasionally causes discomfort and could alter normal walking.

There are two anatomical approaches for inserting intramuscular EMG electrodes into the TP muscle belly: *(i) the posterior-medial*; and *(ii) the anterior insertion*. A video demonstration of both approaches can be viewed via downloadable supplements (see Additional files 1 and 2). The *posterior insertion *involves guiding the electrode posterior to the tibia at a distance mid-way between the ankle and tibial tuberosity. Penetration of the great saphenous vein and posterior neurovascular bundle should be avoided. The *anterior insertion *involves guiding the electrode through tibialis anterior and the interosseous membrane avoiding the deep anterior neurovascular bundle.

When choosing either the *anterior *or *posterior *insertion approach, the two key issues to consider are safety and dynamic stability of the electrode. Cadaveric and MRI studies have shown the *anterior *approach provides a larger safety window when inserting electrodes, as there is a larger distance between osseous structures and neurovascular bundles compared to the *posterior *approach [25,26]. Through piloting and preparation for previous TP EMG work [12], we have found the *anterior *approach to be unstable during walking. The most frequent problem is retraction of the electrode tips from tibialis posterior through the interosseous membrane into tibialis anterior. Further research is required to quantify the success rate and stability of both the *anterior *and *posterior *insertion techniques under dynamic and non weight bearing conditions.

**Figure 3:**
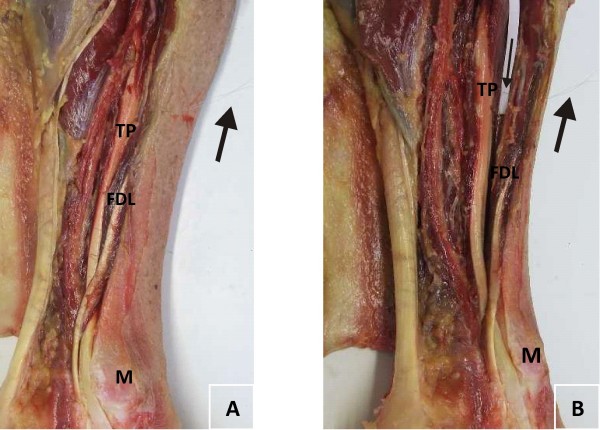
**Audit of placement of intramuscular electrode**. Gross anatomy of dissected limb with intramuscular electrode inserted, indicating; flexor digitorum longus muscle/tendon (FDL), tibialis posterior tendon (TP) and medial malleolus (M). Large arrow indicates wire electrode protruding from limb (3a and 3b) and small arrow indicates wire electrode passing through the muscle belly of flexor digitorum longus and into tibialis posterior (3b) with white paper to highlight electrode.

Historically, intramuscular insertion procedures were undertaken blindly without the aid of current imaging techniques. Recent advances in imaging have improved the accuracy of intramuscular electrode insertions with the use of ultrasound to visualise the target zone and key structures. Ultrasound imaging facilitates real-time observation of the insertion and identification of the neurovascular bundle and anatomical variants. A recent investigation of TP intramuscular electrode insertion, via the posterior approach, was undertaken in five fresh frozen cadaver limbs (RS) with the use of ultrasound guidance. All five electrodes were correctly located in the muscle belly of TP; figure 3 illustrates an example of one dissected fresh frozen cadaver limb and the intramuscular electrode.

Experience gained (GSM) in performing more than 150 intramuscular EMG electrode insertions into TP has led to some important practical insights. Participants usually describe low to mild discomfort during the insertion procedure with approximately 1 in 20 describing severe pain, although this has not been quantified using a validated pain scale. When participants experience severe pain, the wires are removed and a second attempt at relocating new wire electrodes is undertaken; rarely is a third attempt required. During walking, participants usually describe 'mild' pain for the first couple of minutes, which frequently subsides to 'no' or 'low' pain after this period. Mild calf pain is often experienced for 24 hours following the insertion procedure. There were no reported cases of serious complications such as infection. The use of wire electrodes is generally a safe and effective method of investigating tibialis posterior EMG during walking.

### Tibialis posterior EMG in health and disease

**Figure 4:**
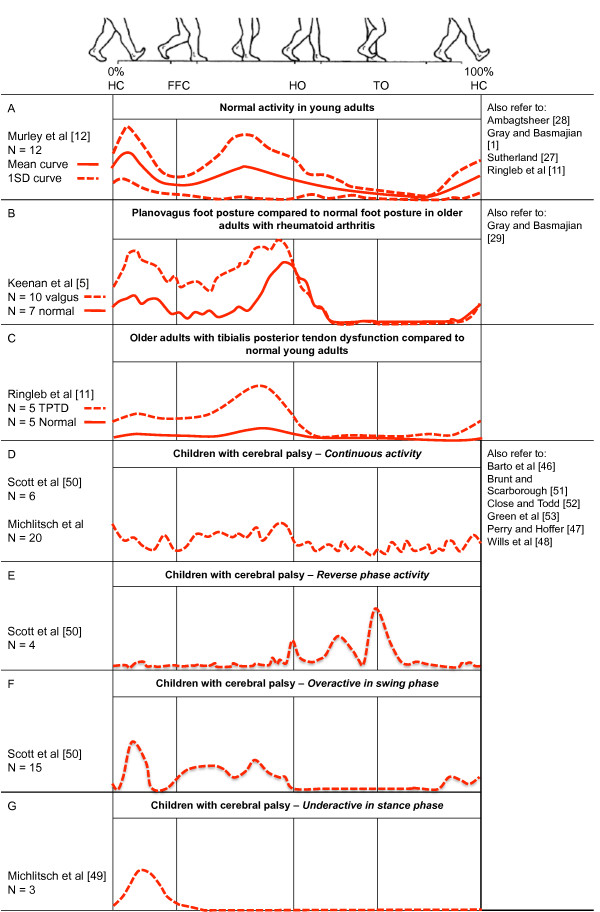
**Tibialis posterior EMG activity during walking in health and disease**. Tibialis posterior EMG activity during walking in health and disease – schematic estimates for ensemble-averaged tracings adapted from the respective studies. 0% and 100% represents heel contact to ipsilateral heel contact. Vertical lines show average timing of temporal gait events. Time resolution is approximated from original work to show a single gait cycle during walking. Amplitude characteristic are not scaled and cannot be compared among different studies. Linear envelopes for figure D-G show estimated unfiltered/unrectified signals. NB. Where multiple studies are available for each category, representation was based on the most recent work with the largest sample size.

Current literature has characterised TP EMG during gait among normal and pathological populations and with various interventions including antipronation taping, foot orthoses and athletic footwear. Figure 4 summarises TP EMG profiles during walking among these populations.

#### Normative TP EMG during walking and running

Normative EMG for TP during walking is based on studies with typically small sample sizes (ranging from 5 to 12) and with participants' age ranging from 18 to 76 years [5,11,12,27-29]. These studies have reported normal TP EMG activity to occur during the stance phase of walking in both young and older adults, with low-level activity in late swing phase. Early studies reported varied periods of TP EMG activity [27-29]; however, without the use of current imaging techniques such as ultrasound, the accuracy of intramuscular electrode placement is unclear. More recent studies report TP activity as bi-phasic, with activity occurring during contact and either midstance or propulsive phases of gait [5,11,12] (Figure 4a). Tibialis posterior EMG is characterised by high between participant variability among healthy adults during walking. Average TP EMG amplitude during walking is estimated to be approximately 20–25% (standard deviation 10–15%) when normalised by a maximum isometric reference contraction [12].

TP EMG activity during running was characterised by Reber and colleagues [30] when they compared three running paces in fifteen recreational runners (mean age: 26 years). During the shortened period of stance phase observed in running, TP displayed a single burst at all three paces at an amplitude of approximately 70–80% (normalised by what the authors described as a 'manual muscle test'). For the fastest running speed, TP displayed a second burst during mid-swing phase.

Overall, the availability of normative EMG for TP during walking is based on relatively small sample sizes and is limited to only adult and older adult participants. Despite the absence of normative data, other studies have investigated TP EMG activation with pathological conditions including rheumatological and neurogenic diseases. With the high variability seen in healthy people, it is difficult to conclude whether the findings from studies investigating abnormal muscle activity are meaningful.

#### Tibialis posterior tendon dysfunction

Tibialis posterior tendon dysfunction (TPTD) has been reported as the most common cause of adult acquired flatfoot [8,22,31,32] yet the aetiology of TPTD and the causal relationship between flatfoot and TPTD remains unclear [33-36]. Whilst numerous studies have investigated the surgical management of this condition, including histological examination of the tendon, only one study has investigated TP muscle function in TPTD [11]. This study reported TP EMG in five female participants with acute stage II TPTD (mean age: 69 years) compared to five healthy adult volunteers (mean age: 27 years). They reported significantly greater TP EMG amplitude in participants with TPTD during the second half of stance phase compared to the control group (Figure 4c). Significant differences in muscle activation were also reported for other lower limb muscles and it was postulated that these differences were an attempt to minimise the acquired flatfoot deformity [11]. Whilst this study has provided important preliminary evidence in terms of TP function; the findings are limited by the small sample size and the results were expressed relative to a maximum voluntary contraction which may have been influenced by patient symptoms [37].

#### Rheumatological disease

It has been suggested that certain rheumatological conditions may predispose to TPTD including rheumatoid arthritis (RA) [38,39] and seronegative inflammatory disease [4,40]. In patients with RA, TPTD has a reported prevalence between 13–64% dependant upon the diagnostic criteria employed [39]. In an RA population, TPTD is frequently associated with a pes plano valgus deformity yet the relationship between the two remains ambiguous. Some authors speculate that tenosynovitis and an attenuated tendon is the cause of the valgus hindfoot [41] whilst others hypothesise that subtalar and midfoot abnormalities are more likely to be the cause [3,42]. Further theories include stress related mechanical alterations resulting from soft tissue changes and instability [42,43], whilst others cite increased pronation forces as the cause and report an association with genu valgum [5].

Despite the uncertainty regarding the pathogenesis of pes plano valgus, gait analysis has been shown to improve our understanding of this condition in RA [44,45]. Yet little is known regarding TP function in this patient group with only one paper investigating TP EMG in an RA population with established disease (mean disease duration 25 years, range 5–50 years in the valgus group). Utilising intramuscular EMG, Keenan and colleagues [5] demonstrated increased TP EMG amplitude in ten patients with RA and a valgus hindfoot alignment compared to seven control subjects with RA and normal foot posture (Figure 4b). It was hypothesised that the increased activity was an attempt to support the collapsing MLA. Whilst these findings indicate a similar trend to those of Ringleb and colleagues [11] in a TPTD population, further work is required from a larger sample size.

#### Neurological disease with focus on cerebral palsy

TP muscle dysfunction is likely to occur with many neurogenic conditions, however little is known about how many of these conditions affect TP muscle activity during walking. Cerebral palsy is one neurogenic condition where TP muscle activation has been investigated as part of several laboratory-based clinical assessments [46-53]. Cerebral palsy frequently causes varus or equinovarus foot deformity which can be painful and disabling, often resulting in surgical correction.

Intramuscular electrodes have been utilised to assess TP muscle activation among infants, children and young adult patients (age range: 4–24 years) – often as part of a surgical planning procedure (Figure 4d–g) [46-50]. Among these studies, TP muscle dysfunction is reported to include; (i) an active 'out of phase burst' (i.e. greater activity during swing phase compared to stance phase), and (ii) a continuous burst throughout the gait cycle [48]. TP dysfunction has also been reported as 'overactivity in swing phase' – characterised by a period of low-level TP EMG activity prior to heel contact [50,51]. However, more recent EMG data from a young-adult population indicates that low-level pre-heel strike activation of TP is normal [12]. Of the twenty-five cases presented by Scott and Scarborough [50], fifteen were classified as having 'overactivity in swing phase' and eight of these cases displayed 'phasic' (i.e. normal) tibialis anterior activity. Therefore, it appears likely that eight of the twenty-five cases referred for split TP transfer surgery actually displayed normal tibialis posterior and tibialis anterior EMG during walking. It is noted these patients also displayed continuous gastrocnemius overactivity, which may provide further explanation regarding the cause of the varus or equinovarus deformities.

A further study by Michlitsch and colleagues [49] involved a retrospective study from pre-operative data recorded from seventy-eight patients assessed over an 11-year period. They reported approximately 1/3 of varus deformities linked with cerebral palsy are associated with TP alone and a further 1/3 are associated with abnormal activity from a combination of abnormal TP and tibialis anterior muscle dysfunction. One subtype of TP dysfunction was described as 'under-activity' characterised by a single burst during contact period. Again, more recent EMG data from a young adult population shows a single burst from TP occurring during only contact period is normal [12].

While there is consensus among these investigations that both TP and tibialis anterior contribute to varus or equinovarus foot deformity with cerebral palsy, one major shortcoming is that none of these investigations have directly compared TP EMG profiles to age matched control groups within the same study. This may account for the different and potentially invalid classifications among these studies of TP dysfunction with cerebral palsy. Further normative EMG from TP is required to inform studies investigating pathological sub-types of TP muscle dysfunction in children with cerebral palsy.

### Tibialis posterior response to intervention

#### Foot orthoses, antipronation taping and footwear

Only one study has investigated the effect of foot orthoses on TP activation during walking [54] despite foot orthoses being the mainstay of conservative intervention for early-stage TPTD. Intramuscular TP activity was recorded from five participants (age range: 25–69 years) with flat-arched foot posture using three different styles of foot orthoses. This study found no systematic changes in TP EMG with the three types of foot orthoses. A similar result was reported for another study investigating three styles of athletic footwear, each with a custom-made midsole aimed at inducing foot pronation and supination during running [9]. This study investigated TP EMG amplitude and temporal characteristics in 10 males (average age: 27 years), however no significant changes were reported for TP EMG among the three shoe styles.

These findings contrast another investigation on the effect of anti-pronation taping on TP EMG during walking in four young- to middle-aged adults with flat-arched feet [55]. Franettovich and colleagues [55] reported a systematic decrease in average and peak TP EMG amplitude during the midstance/propulsive phases of between 21–45%, compared to baseline (barefoot walking). Conservative physical therapies such as foot orthoses, antipronation taping and footwear are considered to perform an important function in altering TP muscle activity during walking, particularly with individuals that have flat-arched foot posture. While there is some preliminary evidence regarding the effect of antipronation tape on TP EMG muscle function [55], the available literature comprises only one investigation based on four participants.

## Conclusion and future recommendations

A number of studies investigating TP EMG activation in health and disease have been undertaken with small sample sizes providing preliminary evidence of either abnormal function or response to intervention. Accordingly, further EMG studies, recruiting larger sample sizes and representation from the younger and older populations, are required to investigate both the effect of interventions on TP muscle activity and to establish a reference database. Whilst it has been recognised that TP plays a vital role during gait, further work is required to more fully understand the role of TP in the development of pathology and in disease-specific populations including RA, cerebral palsy and TPTD.

In summary, TP EMG remains a specialist investigation undertaken in relatively few centres internationally; however, this technique has multiple applications both in research and in planning interventions and evaluating outcomes. Recent advances in technology, including imaging, represent an opportunity to employ this technique more frequently and advance our understanding in a variety of areas.

## Competing interests

The authors declare that they have no competing interests.

## Authors' contributions

DET and JW conceived the idea for the review, RS and GSM drafted the manuscript and the figures, GSM prepared the video supplement, JW and DET critically revised the manuscript. All authors read and approved the final manuscript.

## Supplementary Material

Additional file 1 **Posterior approach**. A video demonstration of the posterior approach of intramuscular electrode insertion. Additional files 1 and 2 can only be viewed using the latest version of QuickTime Player which can be downloaded via the following link: http://www.apple.com/downloads/ Click here for file

Additional file 2 **Anterior approach**. A video demonstration of the anterior approach of intramuscular electrode insertion. Click here for file
